# Frequency of Asymptomatic Deep Vein Thrombosis in Hospitalized Patients With Acute Exacerbation of Chronic Obstructive Pulmonary Disease (COPD)

**DOI:** 10.7759/cureus.69858

**Published:** 2024-09-21

**Authors:** Ishfaq Ahmed, Kamran Khan, Nousheen Akhter, Syed Amanullah Shah, Syed Hidayatullah, Dimple Chawla

**Affiliations:** 1 Pulmonology, Fatima Jinnah Institute of Chest Diseases, Quetta, PAK; 2 Pulmonology, Jinnah Postgraduate Medical Centre, Karachi, PAK; 3 Pulmonology, Bahria University Medical and Dental College, Karachi, PAK

**Keywords:** anticoagulation, chronic obstructive pulmonary disease, copd exacerbation, copd mortality, deep vein thrombosis, pulmonary embolism

## Abstract

Introduction: Acute exacerbations of chronic obstructive pulmonary disease (COPD) are always associated with high mortality. Because of the presence of some concomitant risk factors such as immobilization and bronchial superinfection, patients who are admitted for acute exacerbations of COPD are generally considered to be at moderate risk for the development of venous thromboembolism. Thromboembolism is a cause of unexplained dyspnea, exacerbations, and mortality in COPD. This study aims to determine the frequency of asymptomatic deep vein thrombosis (DVT) in patients with acute exacerbations of COPD presented at a tertiary care hospital.

Methods: This is a descriptive cross-sectional study conducted at the Department of Chest Medicine, Jinnah Postgraduate Medical Center (JPMC), Karachi. A duplex ultrasound study of both lower limbs was performed by a sonologist to assess asymptomatic DVT in patients with acute exacerbations of COPD.

Results: The mean age of the sample was 59.64 ± 9.711 years. Out of 106 patients, 95 (90%) were male and 11 (10%) were female. Asymptomatic DVT was found in 16 (15%) patients with acute exacerbations of COPD. Male patients exhibited a higher incidence of DVT, with 12 cases versus four in females, a statistically significant finding (p=0.03). Additionally, DVT was significantly more prevalent among patients with restricted mobility, with all 16 cases occurring in this group (p=0.006). Age did not show a statistically significant difference in DVT occurrence between patients above and below 59 years.

Conclusions: Deep venous thrombosis is a common occurrence in COPD exacerbations. It is a risk factor for pulmonary embolism that carries a high mortality. All patients with COPD exacerbations may need to be assessed for thromboembolic events. COPD morbidity and mortality are continually rising despite efforts to recognize new phenotypes and treatments. The cause may be the association of unrecognized and untreated thromboembolism. Prompt diagnosis and treatment with anticoagulants in COPD patients may lead to a better prognosis.

## Introduction

Chronic obstructive pulmonary disease (COPD) is a preventable and treatable progressive disease state characterized by airflow limitation due to a fixed or partially reversible airflow obstruction [1]. It is a leading cause of morbidity and mortality worldwide and results in an increasing and substantial economic and social burden. COPD is the fourth most common cause of death worldwide and is estimated to be the third leading cause of death [2]. It has a prevalence of about 10% in adults over the age of 40 years [3]. COPD is a complex disease with multiple systemic comorbidities and complications [4]. Acute exacerbations of COPD are always associated with high mortality [5]. Because of the presence of some concomitant risk factors such as immobilization and bronchial superinfection, patients who are admitted for acute exacerbations of COPD are generally considered to be at moderate risk for the development of venous thromboembolism [6-7]. Earlier estimates suggest that 50% to 70% of all exacerbations of COPD are precipitated by an infectious process, while 10% are due to environmental pollution; up to 30% of exacerbations are caused by an unknown etiology [8-9]. Thus, the cause of acute exacerbations of COPD is often difficult to determine [10]. The prevalence of DVT in COPD patients requiring hospitalization in the West is generally accepted to be in the range of 12% to 15%, although some studies have reported rates greater than 20% [11]. Extensive clinical and post-mortem studies have established that more than 90% of pulmonary embolism originates from venous thrombosis in the deep veins of the lower limbs [12]. And COPD patients are prone to having decreased mobility, a risk factor for thrombus formation.

COPD and deep vein thrombosis (DVT) both have a negative impact on the disease-specific health status. Several studies showed variable frequencies [12], stating that 16% [13], 9.7% [14], and 10.5% [15] of COPD patients were found to have DVT. These findings have yet to be replicated in a local setting, which can potentially offer new information to clinicians and, therefore, influence clinical practice and outcomes of patients with acute exacerbations of COPD. Moreover, data from this study will aid in designing more thorough treatment plans and incorporating regular DVT screenings of COPD patients to prevent disease exacerbations or complications like pulmonary embolism.

This study aims to determine the frequency of asymptomatic DVT in patients with acute exacerbations of COPD presented at a tertiary care hospital.

## Materials and methods

This is a descriptive cross-sectional study conducted at the Department of Chest Medicine, Jinnah Postgraduate Medical Center (JPMC), Karachi, from December 2016 to June 2017. Non-probability consecutive sampling was done to recruit the study participants.

The inclusion criteria for our study were specifically designed to select a representative cohort of patients. We included individuals admitted with acute exacerbations of COPD, encompassing all genders, within the age range of 40 to 60 years. Patients were excluded if they did not provide consent to participate, if they had a history of pulmonary embolism or malignancy, including those currently undergoing treatment for cancer, or if they had active symptoms or signs of DVT (unilateral leg swelling, pain, or tenderness). Patients with a history of stroke, congestive heart failure, chronic liver disease, ischemic heart disease, or those suffering from connective tissue diseases or other autoimmune disorders were also not included in the study. Mobility restrictions were also documented. Those patients who had baseline grade III or IV dyspnea according to the modified Medical Research Council Scale before the exacerbation were categorized as having restricted mobility. These criteria were set to minimize confounding factors and to ensure a more homogeneous study population, focused on the primary objective of assessing the frequency of DVT in patients with acute exacerbations of COPD.

Permission from the Institutional Review Board of JPMC was taken prior to the conduction of the study (approval number: F.2-81/2014-GEN/8098/JPMC, dated November 6, 2014). Written, informed consent was obtained from all the patients included in the study. Demographic information was entered on pre-structured proforma. A duplex ultrasound study of both lower limbs was performed by a sonologist with more than five years of experience in the presence of a researcher for all patients enrolled in the study within 48 hours of admission.

Data was analyzed on SPSS Statistics version 20 (IBM Corp. Released 2011. IBM SPSS Statistics for Windows, Version 20.0. Armonk, NY: IBM Corp.). Means and standard deviations were calculated for the quantitative variables like age, BMI, and duration of COPD. Frequencies and percentages were calculated for the qualitative variables like gender, socioeconomic status, occupational status, immobile time, and DVT. Effect modifiers were controlled through stratification of age, gender, BMI, duration of COPD, educational status, socioeconomic status, occupational status, and immobile time to see the effect of these on outcome variables. A post-stratification chi-square test was applied, taking p<0.05 as statistically significant.

## Results

A total of 106 patients were included to assess the frequency of DVT in patients with acute exacerbations of COPD. Demographic details are shown in Table 1.

**Table 1:** Demographic details of the study participants SD: standard deviation, BMI: body mass index, COPD: chronic obstructive pulmonary disease

Variables	Measures
Age in years (mean ± SD)	59.64 ± 9.71
BMI in kg/m^2^ (mean ± SD)	22.43 ± 4.01
Gender (%)	
Male	95 (90)
Female	11 (10)
Duration of COPD in years (mean ± SD)	4.96 ± 4.07

In the stratified analysis, significant associations with DVT occurrence were noted in relation to gender and mobility. Male patients exhibited a higher incidence of DVT, with 12 cases versus four in females, a statistically significant finding (p=0.03). Additionally, DVT was significantly more prevalent among patients with restricted mobility, with all 16 cases occurring in this group (p=0.006). Age did not show a statistically significant difference in DVT occurrence between patients above and below 59 years. These findings are detailed in Table 2.

**Table 2:** Stratification of DVT in COPD patients with respect to effect modifiers DVT: deep vein thrombosis, BMI: body mass index, COPD: chronic obstructive pulmonary disease

Variable		DVT		p-value
		Yes	No	
Age (years)	30-59	7	38	0.9
	>59	9	52	
Gender	Male	12	83	0.03
	Female	4	7	
BMI (kg/m^2^)	15-23	6	56	0.06
	>23	10	34	
Duration of COPD (years)	1-5	12	64	0.5
	>5	4	26	
Mobility	Restricted	16	63	0.006
	Not restricted	0	27	

## Discussion

We investigated the frequency of asymptomatic DVT in patients with acute exacerbations of COPD. Fifteen percent of patients included in our study had underlying DVT as a triggering factor for exacerbation or a complication. COPD patients experience on average two to four acute exacerbations per year [16]. Each acute exacerbation is accepted as an important mortality and morbidity cause in these patients. The incidence of pulmonary embolus as a reason for acute exacerbations is unknown. The high rate of pulmonary embolism is a well-known reality in DVT-positive cases [17].

A recent meta-analysis showed an overall 20% prevalence of pulmonary embolism in COPD exacerbations [18]. DVT is a less frequently monitored issue in COPD exacerbations, and its prevalence is reported by some studies to be 1.6-12.7% in COPD exacerbations [19-22]. Huisman et al. highlighted the clinical relevance of DVT, noting that a significant proportion of patients (43.5%) with DVT in the lower extremities but no clinical indicators of embolism were found to have a high probability of pulmonary embolism using pulmonary perfusion scintigraphy [23]. They argued for the necessity of investigating pulmonary embolism as a causative factor in the acute exacerbations of COPD, irrespective of clinical suspicion. In intrinsic lung diseases, like COPD, the detection of DVT using compression duplex ultrasound is more reliable than identifying pulmonary embolism with ventilation/perfusion scintigraphy, since the latter is often confounded by COPD-related lung changes that can either mimic or mask the signs of pulmonary embolism, thus increasing misdiagnosis risk [24]. Given that both DVT and pulmonary embolism require similar treatment approaches and that ultrasound has a lower rate of false-positive results, we advocate for ultrasound as the primary diagnostic tool for suspected venous thromboembolism in this patient group.

Our study revealed significant associations of DVT with gender and mobility, underscoring potential risk disparities. Males exhibited a higher DVT prevalence (12 cases) compared to females (four cases), a statistically significant difference (p=0.03), despite a smaller comparison group of females without DVT (7) relative to males without DVT (83). Moreover, restricted mobility markedly correlated with DVT, with all cases (16) occurring in this group and no DVT cases in those without mobility restrictions (p=0.006), suggesting the critical role of mobility in DVT risk. Although a higher proportion of DVT was observed in patients over 59 years, this was not statistically significant, contrasting with the findings of Winter et al. [17], who identified age >59 years as a risk factor. This highlights the importance of considering both gender and mobility in DVT risk assessment and prevention strategies. The minimum BMI was 15 kg/m^2^, while the maximum BMI was 33 kg/m^2^. The difference between the two groups was not found to be statistically significant.

The main limitations of our study are the cross-sectional design, small sample size, and lack of follow-up. This study was a single-center study; hence, the findings do not reflect the true frequency and severity of the disease. Our sample was predominantly male and did not have a control group without acute exacerbations or a healthy group. There is also a need to identify whether thrombus is a complication of COPD exacerbations or a triggering factor. COPD patients are prone to venous thromboembolism because of immobilization, increased systemic inflammation, cigarette smoking, and venous stasis. However, it remains underdiagnosed in these patients because its symptoms mimic COPD exacerbations. Furthermore, thrombus itself may lead to exacerbations, where other identifiable causes of exacerbations are not found [25]. Longitudinal controlled multi-center studies with larger sample sizes are needed to assess causality, mortality, and incidence of pulmonary embolism in patients of COPD who developed DVT.

## Conclusions

Deep venous thrombosis is a common occurrence in COPD exacerbations. It is a risk factor for pulmonary embolism that carries a high mortality. All patients with COPD exacerbations may need to be assessed for thromboembolic events.

COPD morbidity and mortality are continually rising despite efforts to recognize new phenotypes and treatments. The cause may be the association of unrecognized and untreated thromboembolism. Prompt diagnosis and treatment with anticoagulants in COPD patients may lead to a better prognosis.
